# Infective Endocarditis Requiring Mitral Valve Replacement During Second Trimester of Pregnancy

**DOI:** 10.1016/j.jaccas.2024.102344

**Published:** 2024-04-22

**Authors:** Elena Jost, Laura Remmersmann, Miriam Silaschi, Farhad Bakhtiary, Ingo Heinze, Julian Luetkens, Tiyasha H. Ayub, Brigitte Strizek, Waltraut M. Merz, Philipp Kosian

**Affiliations:** a Department of Obstetrics and Prenatal Medicine, University Hospital Bonn, Bonn, Germany; b Department of Cardiac Surgery, University Hospital Bonn, Bonn, Germany; c Department of Anesthesia and Intensive Care, University Hospital Bonn, Bonn, Germany; d Department of Radiology, University Hospital Bonn, Bonn, Germany

**Keywords:** cardiopulmonary bypass, infective endocarditis, pregnancy

## Abstract

Infective endocarditis requiring mitral valve replacement during pregnancy is a rare event. We present a case of infective endocarditis of the mitral valve during second trimester and report maternal and perinatal outcomes. Prompt identification and interdisciplinary treatment is crucial; maternal and fetal follow-up including serial fetal neurosonography is recommended.

## Graphical Abstract

**Figure:**
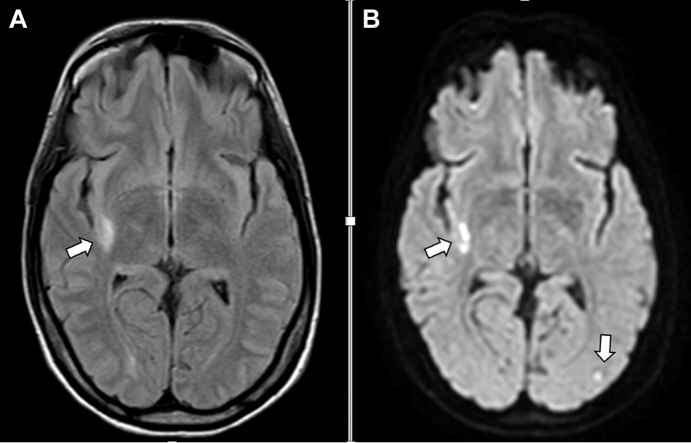

## History of Presentation

A 33-year-old gravida (G) 2 para (P) 1 (one full-term vaginal delivery) with suspected infective endocarditis (IE) of the mitral valve (MV) was referred to the intensive care unit of our institution at 17+0 weeks of gestation (WoG). The patient had presented at a district hospital 5 days earlier with severe headache, difficulties moving around and finding words, as well as rapidly deteriorating general condition. Informed consent was obtained from the patient.

**Learning Objectives**

• To recall that symptoms such as shortness of breath and fatigue are common during pregnancy and may be mistaken for trivial complaints. A thorough history-taking and detailed clinical examination is therefore crucial, and further investigations need to be performed without delay.
• To be aware that a multidisciplinary approach in pregnant patients with IE is essential.
• To recognize that CPB during pregnancy may affect fetal development. Close follow-up including neurosonography should therefore be considered.

## Past Medical History

Her past medical history was non-contributory and no predisposing risk factors were present.

## Differential Diagnosis

Differential diagnoses considered were atrial fibrillation with a consecutive cerebral embolic event, meningitis, and influenza.

## Investigations

Mobile vegetations at the basal posterior MV leaflet (20 × 13 mm) were observed on transesophageal echocardiography (TEE), multiple cerebral microembolic infarctions were found using cerebral magnetic resonance imaging (MRI), and a positive blood culture (methicillin-sensitive staphylococcus aureus) led to the diagnosis of IE.

## Management

**Figure 1:**
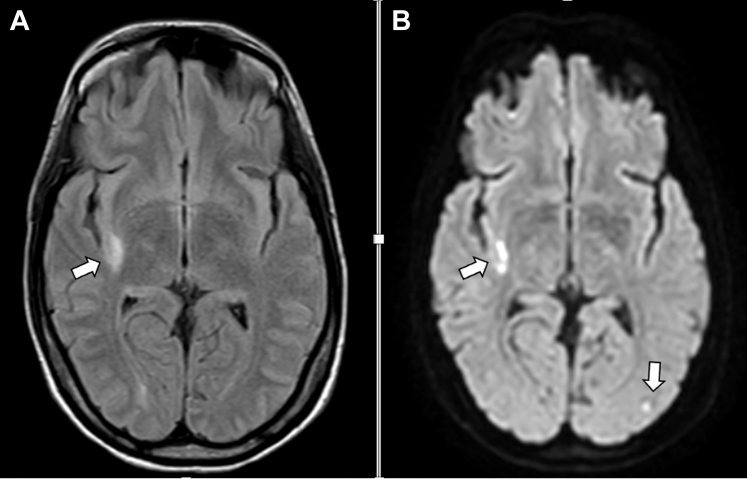
Maternal Brain Magnetic Resonance Imaging Brain magnetic resonance imaging of the patient showing acute stroke lesions due to cerebral embolism in different vascular territories. Embolic lesions are visible on axial (A) fluid attenuated inversion recovery images and (B) diffusion-weighted imaging.

**Figure 2:**
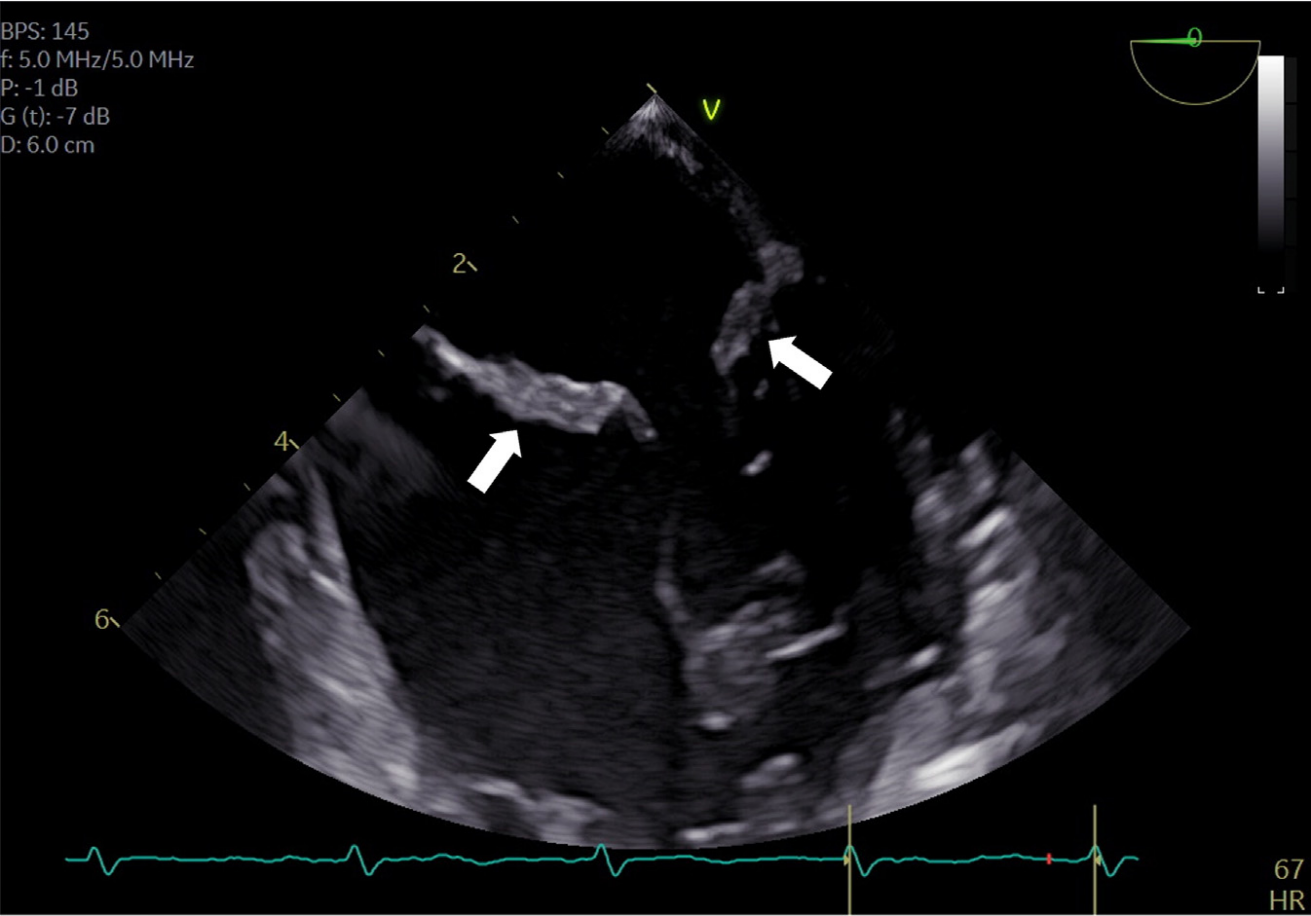
Transesophageal Echocardiogram Transesophageal echocardiography showing vegetations and destruction of the anterior and posterior MV leaflet (20 mm x 13 mm).

**Figure 3:**
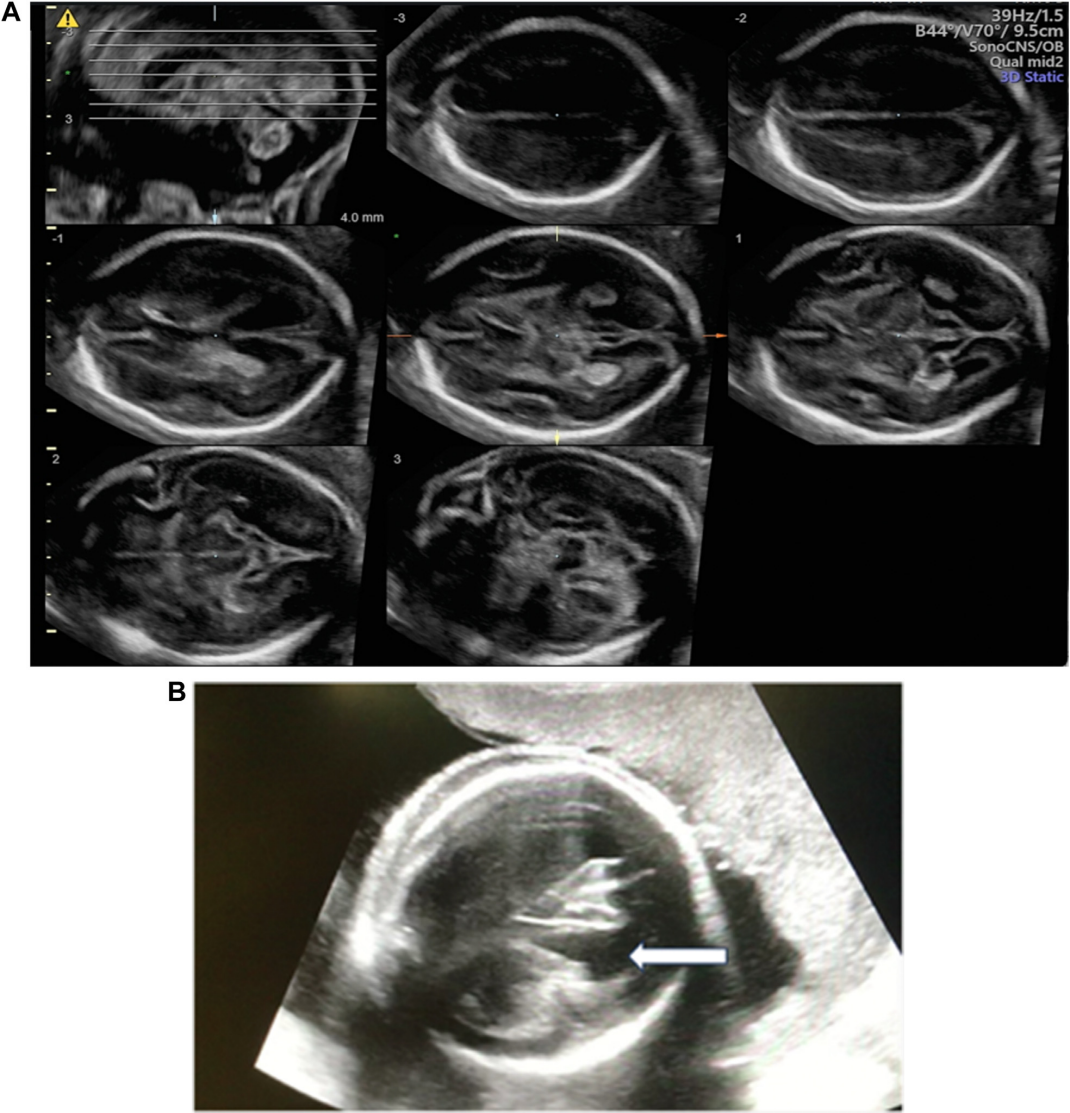
Fetal Neurosonography (A) Fetal neurosonography in transverse planes showing normal findings in our case at 23+0 weeks of gestation. (B) For comparison, an exemplary case of fetal ventriculomegaly (white arrow) due to hypotension and ischemia in a patient on extracorporeal membrane oxygenation during severe acute respiratory syndrome coronavirus 2 infection; transverse plane, 23+5 weeks of gestation.

High-dose parenteral antibiotic therapy with flucloxacillin was initiated, but the patient’s condition deteriorated. Repeat cerebral MRI was performed showing an increase in the number of microembolic infarctions (Figure 1) and on TEE, destruction of the anterior and posterior MV leaflet on TEE was shown (Figure 2 and Video 1). Therefore, a joint decision for surgical treatment was determined in 17+1 WoG. After median sternotomy, cardiopulmonary bypass (CPB) was performed in normothermia. Cannulation was central (aorto-bicaval) to avoid retrograde perfusion. After cardioplegic arrest was induced, the MV was inspected; a severely infected MV with an abscess cavity at the level of P1-P2 reaching into the myocardium of the left ventricle and tissue destruction of both leaflets was found. The MV was beyond repair; therefore, biologic MV replacement was performed which was an intentional decision due to the ongoing pregnancy (Video 2). Aortic cross clamp time was 65 minutes. On postoperative day 8, sinus tachycardia became evident, pericardial effusion was detected in transthoracic echocardiogram, and pericardiocentesis was performed. After persistent sinus tachycardia and grade 1 diastolic dysfunction were discovered, beta-blocker therapy with metoprolol was initiated. Parenteral antibiotic therapy was administered for 6 weeks, as well as therapeutic anticoagulation initially with unfractionated heparin followed by low molecular weight heparin for 3 months. Six weeks after surgery, the patient was transferred for rehabilitation. Regular obstetric, prenatal, and cardiac follow-up was performed, including serial fetal neurosonographic examinations (Figure 3A). Further maternal or fetal complications did not occur.

At 32+0 WoG, the patient presented with preterm premature rupture of membranes. Prophylaxis for neonatal respiratory distress syndrome with betamethasone, tocolytic therapy with atosiban, and antibiotic treatment with cefuroxime and azithromycin were initiated. After interdisciplinary discussion, tocolytic treatment was discontinued to reduce the period of increased risk of infection. A healthy preterm male was born after uncomplicated labor (1,900 g, 46th percentile, UA-pH 7.33, Apgar score 8/9/10) in 32+1 WoG. The patient required curettage for incomplete placenta (estimated blood loss, 800 mL). Postpartum antibiotic treatment with cefuroxime continued for 6 days. After birth, maternal transthoracic echocardiogram did not show any signs of perivalvular leakage; the MV mean pressure gradient was 7 mm Hg. The newborn was transferred to our neonatal intensive care unit. Bilateral pneumothoraces occurred; treatment consisted of drainage and continuous positive airway pressure. Neonatal neurosonography did not detect any abnormalities.

## Discussion

CPB in pregnancy is typically reserved for life-threatening cardiac conditions for patients with unsuccessful conservative treatment. Examples include severe valvular heart disease, aortic dissection, or certain congenital heart defects. A meta-analysis from 2018 reviewing maternal and fetal outcomes in 154 women undergoing CPB between 1990 and 2016 found a mortality rate of 11.2%. Pregnancy loss occurred in 33%, maternal complications in 8.8%, and neonatal complications in 10.8% of cases. The most common maternal complications were persistent heart failure (5.8%), arrythmia (2.2%), and postoperative bleeding (2.7%). Neonatal complications reported were respiratory distress syndrome in 5.2%, developmental delay in 2.6%, and neurologic damage in 2.6% of cases consisting of cortical atrophy, tetraparesis, hydrocephalus, and cerebral anoxia.^1^ Countouris et al^2^ identified 9 patients with IE during pregnancy and up to 6 weeks postpartum. Complications included shock (33%), mechanical ventilation (44%), septic emboli (67%), and noncardiac abscesses (33%). Two patients underwent cardiac surgery on CPB (1 case postpartum and 1 case in 26 WoG) and 2 maternal deaths were reported (septic shock and intracerebral hemorrhage).^2^ In our case, we observed septic emboli as maternal and prematurity and its consequences as neonatal complications.

Monitoring fetal heart rate in viable fetuses (beyond 23 to 24 WoG) should be performed throughout surgery, if possible. The type of monitoring (intermittent or continuous) should be determined on a case-by-case decision, based on type of surgery and gestational age.^3^ Brandstetter et al^4^ suggest serial fetal Doppler ultrasound examinations of the fetal and fetomaternal blood flow also before viability to detect fetal stress caused by hypotension, maternal acidosis, and/or hypercapnia early and to take corrective action. Because of limited data on fetal surveillance during open heart surgery on CPB and its benefits, especially before fetal viability, no standards or guidelines are currently in place. In our case, we decided against fetal monitoring during surgery because of the gestational age.

To increase the chance of fetal survival, CPB adjustments have been proposed. These include the choice of cannulation site avoiding retrograde aortic perfusion, eschewing hypothermia, avoiding hypotension, and maintaining potassium concentrations within normal range.^5^ During general anesthesia for open cardiac surgery on CPB, there is a risk for maternal hypoxia and hypotension, which is highest during transition from corporal to extracorporeal circulation.^4^ Little is known about the effect of these changes on the fetal cerebral perfusion and its sequelae. In term or near-term infants, there is evidence that acute perinatal hypoxia can cause fetal encephalopathy.^6^ In pre-term and pre-viable fetuses, our knowledge about mechanisms leading to brain injury is more limited. Fetal stroke can be caused by ischemic, thrombotic, or hemorrhagic injury.^7^ Mechanisms thought to contribute to fetal brain injury include isolated hypoxia and hypoxia combined with hypercapnia (termed asphyxia). Depending on the severity and gestational age at onset of either one or both of those mechanisms, different patterns of brain injury (ventriculomegaly, periventricular leukomalacia, delay of maturation) might be detected later in the fetus.^8^^,^^9^ In general, in a surviving fetus after an episode of severe maternal hypotension or prolonged hypoxia, brain injury can only be detected with a delay of several weeks. Avoiding maternal hypotension and hypercapnia might prevent fetal brain injury; however, serial ultrasound examinations of the fetal brain after surgery over a period of 6 to 8 weeks are recommended. In case of abnormal findings, detailed counseling of the parents is required. In Figure 3A, normal neurosonographic findings of our case at 23+0 WoG are shown 6 weeks after MV replacement. For comparison, Figure 3B shows fetal ventriculomegaly due to ischemia and hypotension in a patient on extracorporeal membrane oxygenation due to severe acute respiratory syndrome coronavirus 2 infection in 25+5 WoG as an exemplary case.

## Follow-up

At the time of reporting, the newborn (6 months of age) and his mother are in good condition and were both discharged from the hospital.

## Conclusions

Members of the pregnancy heart team including obstetricians, maternal-fetal medicine specialists, cardiologists, cardiothoracic surgeons, infectious disease specialists, anesthesiologists, and neonatologists need to work together. Close maternal and fetal monitoring throughout the remaining pregnancy is essential.

## Funding Support and Author Disclosures

This work was supported by the Open Access Publication Fund of the University of Bonn. The authors have reported that they have no relationships relevant to the contents of this paper to disclose.

## Appendix

Video 1 TEE Showing Vegetations and Destruction of the Anterior and Posterior MV Leaflet

Video 2 TEE After Biologic Mitral Valve Replacement
